# Preparation of an FA-Based Discoloration Material and Its Application in Jewelry Design

**DOI:** 10.3390/ma17225628

**Published:** 2024-11-18

**Authors:** Xiaomin Zhang, Xiangrui Gao, Yue Yuan, Guangqin Yang, Yanchen Li

**Affiliations:** 1 Jewelry Academy, China University of Geosciences, Beijing 100083, China; zhangxiaomin9208@163.com; 2 The Future Laboratory, Tsinghua University, Beijing 100084, China; 3 City Design School, Central Academy of Fine Arts, Beijing 100105, China; gaoxiangrui0515@163.com (X.G.); yuanyue19920911@163.com (Y.Y.); 4 School of Earth Sciences and Resources, China University of Geosciences, Beijing 100083, China; yangguangqin88@163.com; 5 Academy of Arts & Design, Tsinghua University, Beijing 100084, China

**Keywords:** fly ash, industrial waste, material modification, low-carbon design

## Abstract

Fly ash (FA) is the main solid waste emitted from coal-fired power plants. Due to its high yield, low utilization rate, and occupation of a large amount of land, it exerts enormous pressure on the Earth’s environment. With the deepening of the concept of sustainable development, exploring the reuse of industrial waste such as FA has become a key strategy. If FA can be combined with commonly used jewelry in people’s lives, it will be of great significance to promote the high-net-worth utilization of FA. Therefore, this study synthesized a fly-ash-based composite material with color-changing function and combined it with necklaces as the main material. In the first stage, after blending fly ash and slag, an alkaline activator with a total mass of 10% was added. When the proportion of fly ash was 60%, the compressive strength of the prepared fly-ash-based composite material reached 10.1 MPa. This was attributed to the reaction between sodium silicate in the alkaline activator and free CaO, MgO, and other substances in the fly ash to form hydrated silicate colloids, which solidify the fly ash and transform it into a complex three-dimensional network skeleton. In the second stage, a UV resistant coating with thermochromic function was obtained by blending acrylic resin, TiO_2_, and a thermosensitive color-changing agent. It was applied to the surface of fly-ash-based composite materials, and the results showed that as the content of the color-changing agent increased, the number of pores on the surface of the coating gradually decreased. When the content of color-changing agent was 10%, the prepared 10%FAB not only had good surface color but also had good thermal stability, UV absorption ability, superhydrophobicity, and mechanical properties. Therefore, 10%FAB was selected as the basic material for jewelry design. In the third stage, the traditional Chinese technique of “gold inlaid with jade” was utilized to develop jewelry applications for the FA composites. As such, 10%FAB was processed into necklaces, which not only had modern design aesthetics but also had good color-changing effects above 30 °C. And after a long period of UV aging experiments, the necklace did not show any wrinkles, bubbles, or other phenomena. Due to the excitation of TiO_2_ hole–electron pairs, the necklace’s UV absorption ability was further improved. This study demonstrates the potential application of industrial waste in decorative products, expands the high-end utilization of fly ash as a low-cost material, and provides new ideas for building a low-carbon lifestyle.

## 1. Introduction

**Figure 1:**
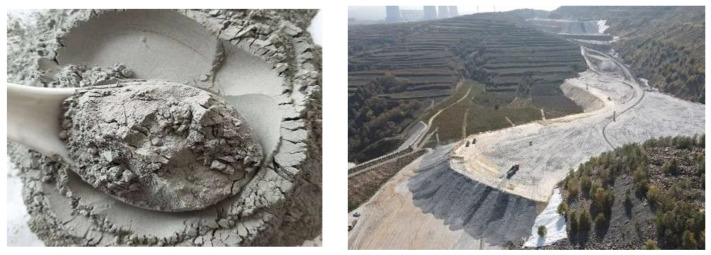
Appearance and disordered storage of FA.

Coal-fired power generation remains a dominant source of electricity worldwide, characterized by its high energy conversion efficiency, low production costs, and well-established technology. However, this process concurrently generates substantial quantities of solid waste, primarily in the form of FA. Global FA production is estimated at approximately 150 million tons annually, with China and India accounting for nearly 70% of this output. The immense volume of FA poses a significant environmental challenge, requiring substantial landfilling and exerting pressure on ecosystems (Figure 1). This issue has heightened the urgency for exploring comprehensive utilization strategies for FA, particularly given the increasing emphasis on carbon emission reduction. Currently, some researchers are paying attention to the comprehensive utilization of fly ash, exploring its applications in building materials, road construction, soil improvement, and water source purification; reducing the exploitation of nonrenewable resources such as mountains; and demonstrating the potential to promote the development of a “low-carbon economy”. However, these applications are mainly based on rough processing and are not closely integrated with people’s daily lives. Jewelry is a common decorative item in people’s lives. Jewelry design has evolved alongside human civilization, traditionally relying on gemstones and precious metals. However, contemporary advancements in materials science and technology, coupled with a growing demand for personalized expressions, have led to the emergence of “non-traditional” materials in jewelry design [1]. If fly ash can be modified to enhance its additional functions and then be applied to jewelry with huge production capacity, it will provide new ideas for the “inferior use” of solid waste and have important significance in promoting global sustainable development [2,3,4,5].

The particle size of FA is generally between 1 and 100 μm. It has a complex composition and is characterized by being highly fine, lightweight, highly porous, and rich in trace elements. Additionally, it exhibits low density, heat resistance, corrosion resistance, plasticity, environmental protection, and resource utilization and has a wide range of potential applications in the fields of building materials, cement manufacturing, soil improvement, and environmental treatment [6,7,8,9,10,11,12,13,14,15]. However, the poor surface activity of FA means it does not easily compound with other materials, and some chemical excitation means are needed to improve its interfacial interaction with other materials. For example, Onoue et al. showed that the presence of alkali exciters and concentration have an important effect on the reaction rate of FA and the formation of geopolymer gels [16]. According to a study by Soutsos et al., when FA was activated by 12.5 M NaOH and cured at 70 °C, its microstructure properties and mechanical strength were improved. When studying the synthesis and properties of high-calcium FA base polymer [17], Kamhangrittirong et al. found that GPC was prepared by using NaOH and Na_2_SiO_3_ as base activators and curing at room temperature for 3~28 d. It was found that the compressive strength could be increased to 30.0 MPa by increasing the ratio of CFA/base activator [18]. Ehsanul Haq et al. found that heat and moisture in GPC altered the mechanical strength to a maximum of 61.4 MPa in the synthesis of FA- and bottom-ash-based matrix polymers using water glass and NaOH as alkali exciters [19]. Mustafa et al. found that the compressive strength increased to 71.0 MPa when the Na_2_SiO_3_/NaOH ratio was 2.5, the curing time was 24–72 h, and the curing temperature was 60–90 °C [20]. Seifan, M et al. studied the physico-mechanical properties of FGPC mortar cured with the addition of silica particles (NS) and microsilica particles (MS), and the 5% NS particles had the best physical properties, with a compressive strength of 22.5 MPa, which not only reduces the resistance to water permeability but also improves the long-term strength of the geopolymer [21]. Li et al. found that, at a relatively low sodium-hydroxide-to-CFA mass ratio of 0.5, effective alkali fusion could be achieved at 350 °C for 0.5 h. Geopolymer pastes were cured at 40 °C for 7 days, reaching a maximum compressive strength of 34.0 MPa [22].

To enhance the mechanical strength of FA-based composites, research has demonstrated the efficacy of employing multi-component precursor systems for gelling agents [23]. The incorporation of FA into alkali-activated slag systems results in the formation of alkali-activated slag–FA cementitious materials, leading to an increase in sodium hydrated silicoaluminate (N-A-S-H) gel formation, which contributes to improved mechanical strength [24]. These findings suggest that alkali activation enhances the viscosity of FA, enabling the production of composites with enhanced mechanical properties when combined with bottom ash and other materials. This provides a foundation for exploring the application of FA in jewelry design.

Therefore, this study took fly ash as the research object, recycled slag as the mechanical reinforcement phase, and sodium silicate as the cementitious material and prepared fly-ash-based composite materials by adjusting the material ratio. In order to solve the problems of poor waterproof property, easy slag falling, and the single color of the fly ash composite, the thermosensitive and color-changing composite was prepared by sol–gel method with acrylic resin as the carrier, and the surface of the fly ash composite was coated with the thermosensitive and color-changing function. The use of color-changing fly ash in jewelry design using the traditional Chinese “gold inlaid jade” technique results in necklaces with excellent UV resistance and color-changing properties. This study provides new ideas for the high-net-value utilization of industrial waste.

## 2. Materials and Methods

### 2.1. Materials

The required chemicals and their correlating uses in this study can be found in Table 1.

### 2.2. Preparation and Design Applications of FA Matrix Composites

#### 2.2.1. Preparation of FA-Based Composites

In the FA and recycled slag co-mingled configuration of the four samples, FA accounted for 60%, 70%, 80%, or 90%, and the four samples were called F6S4, F7S3, F8S2, and F9S1. The total mass of 10% of the alkaline excitant was then added. The solid–liquid ratio of the alkaline excitant was 0.5:1. The mass ratio of sodium hydroxide and sodium silicate in the alkaline excitant was 1: l. Sodium hydroxide occupied the total mass of 10 M. The weighed solid powder and the prepared alkaline exciter were added to a blender and mixed for 10 min. The well-mixed slurry was injected into a 20 mm × 20 mm × 20 mm cubic plastic mold and vibrated for 5 min to reduce the bubble content in the slurry. The samples were taken out and sealed, cured in a drying oven at 60 °C for 24 h to demold, and placed in a sealed bag to be cured in a room temperature environment for 10 d. The samples were named FAB.

#### 2.2.2. Surface Treatment of FA-Based Composites

**Figure 2:**
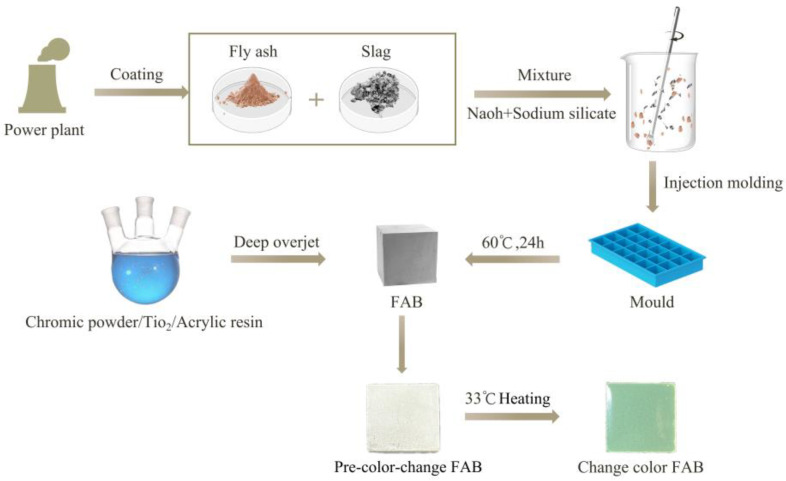
Preparation of FA-based composites and surface treatment.

The acrylic resin was dissolved in acetone at a mass ratio of 1:1 and stirred at room temperature to dissolve the acrylic resin. Titanium dioxide was added at 10% of the mass of the acrylic resin, and the mixture was stirred well to obtain the anti-ultraviolet acrylic resin. The temperature-sensitive color change agent was added to the anti-ultraviolet acrylic resin with a mass ratio of 0%, 5%, 10%, or 20%. The active diluent cyclexone was diluted in a total volume of 1:0.5 and stirred for 2 h at 45 °C, then configured with temperature-sensitive discoloration emulsion. The final samples were named 0%Film, 5%Film, 10%Film, and 20%Film. Finally, the fly ash composite material was impregnated into the discoloration emulsion to coat the emulsion surface for 72 h; these were named 0%FAB, 5%FAB, 10%FAB, and 20%FAB. The main process is shown in Figure 2.

#### 2.2.3. Preparation of Color-Changing FA-Based Necklace

**Figure 3:**
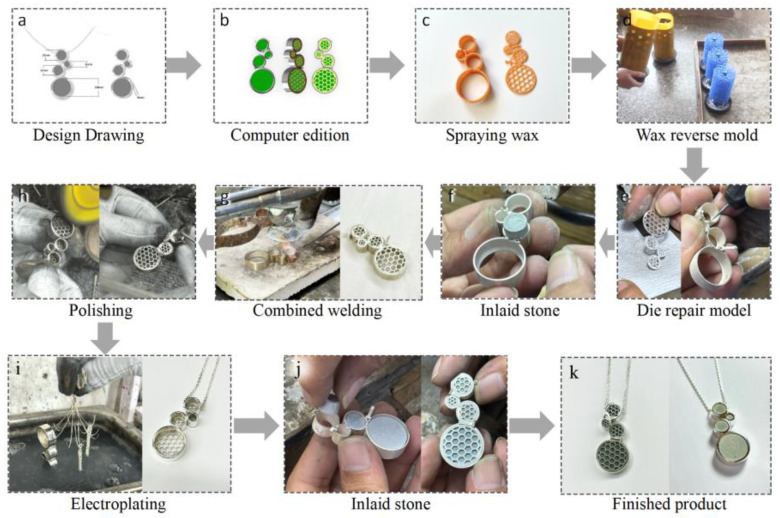
Preparation process of color-changing FA-based necklace of (**a**) Desgn drawing; (**b**) computer edition; (**c**) spraying; (**d**) wax reverse mold; (**e**) die repair model; (**f**) inlaid stone; (**g**) combined welding; (**h**) polishing; (**i**) electroplating; (**j**) inlaid stone; (**k**) finished product.

The main body of the necklace was assembled with four circles made of FAB, with sizes of 10 mm, 6 mm, 10 mm, and 20 mm, all with a thickness of 6 mm. The exterior was inlaid with 925 silver metal, and the main material was inlaid through the structure of the integral buckle. The fly ash base discoloration material was made into a circle of the corresponding size using the mold, and the surface was polished with sandpaper to make it smoother. The external metal was computer modeled according to the design drawing; the metal shell was made through the process of wax spraying, pouring, mold holding, and polishing; and then the obtained circular main material was embedded into the shell. The main process is shown in Figure 3.

### 2.3. Characterization

Scanning electron microscope (SEM, S-4800, Hitachi, Japan) was used to observe the internal microstructure of the samples. The samples were bonded to the sample stage with conductive tape, and their surfaces were sprayed with gold for the observation of the morphology. The scanning voltage was 5.0 kV. Elemental distribution was detected by mapping. The infrared spectra of the microstructural morphology of the samples were tested by a Fourier-transform infrared spectrometer (FTIR, FTIR-7600, Lambda, Australia), with a spectral resolution of 0.4 cm^−1^ and a scanning range of 400–4000 cm^−1^. The samples were analyzed by a thermal loss of gravity analyzer (TGA, TGA209F3, NETZSCH, Germany). The samples were thermogravimetrically analyzed using a thermogravimetric analyzer (TGA, TGA209F3, Germany) with a temperature range of 30–800 °C, a test rate of 10 °C/min, and a test atmosphere of N_2_. A UV-visible spectrophotometer was used to analyze the transmittance of the samples. The scanning range was 200–800 nm, and the hydrophobicity of the samples was detected by the water contact angle test.

## 3. Results and Discussion

### 3.1. Performance Analysis of FA-Based Composites

**Figure 4:**
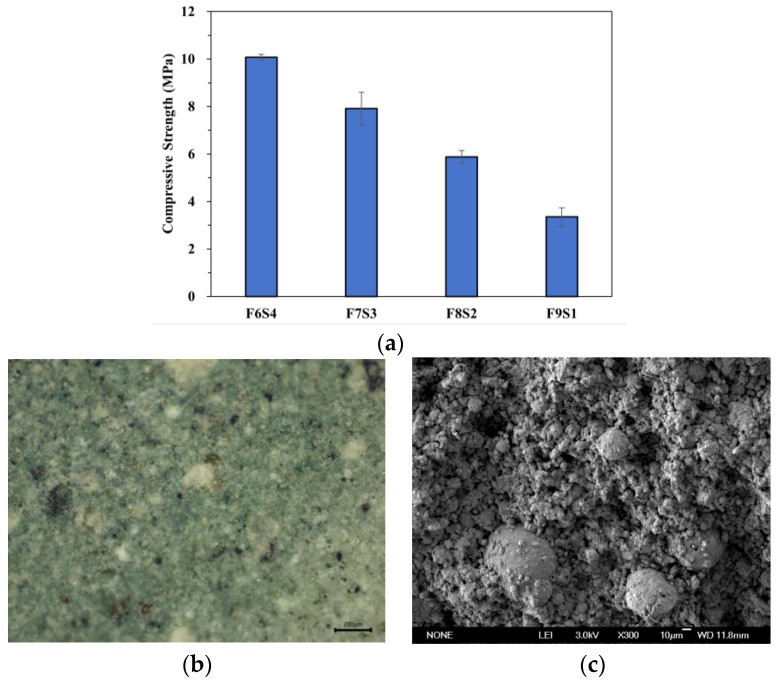
(**a**) Compressive properties of FA-based composites; (**b**) micro-morphology of FA-based composites; (**c**) gelation effect of FA-based composites.

After the specimens reached the age of curing, the dimensional stability of the samples with different ratios was compared by performing compressive tests on the FA-based composites. As can be seen from Figure 4a, the compressive strengths of F6S4, F7S3, F8S2, and F9S1 are 10.1 MPa, 7.9 MPa, 5.9 MPa, and 3.4 MPa, respectively, and the results indicate that the compressive strength of the composites decreased with the increase in the proportion of FA, and slag was the key factor in enhancing the dimensional stability of the composites. This may be attributed to the fact that an increase in slag leads to a decrease in the amount of exciter, leading to an increase in the solids content in the slurry and an increase in the viscosity, which in turn leads to denser bonding between the components of the composites. Conversely, with an increase in FA content, the water absorption of the composite increases and the viscosity decreases, which leads to a decrease in the internal component bonding. Therefore, F6S4 is screened as the substrate for the preparation of FA-based necklaces. Figure 4b shows the micrograph of F6S4, with TiO_2_ appearing whiter, slag as black spots, and fly ash in the gray area. Several different materials are tightly combined and uniformly dispersed, but TiO_2_ exhibits a certain degree of agglomeration phenomenon. From the SEM image (Figure 4c), it can be seen that the fly ash particles aggregate together to form a block-like structure, and the larger particles distributed in the block-like material may be slag and agglomerated TiO_2_. Different materials are closely combined and interwoven, which may be attributed to the reaction of sodium silicate with the active ingredients in the fly ash to generate gel such as sodium aluminosilicate and hydrated calcium silicate. These gels form compact structures during the hardening process, thus improving the strength and durability of the materials. Sodium silicate can also react with free CaO, MgO, and other substances in fly ash to form hydrated silicate colloids, thereby solidifying fly ash and transforming it into a complex three-dimensional network skeleton of larger particles in block-like materials. Fly ash also has a filling effect, which can not only fill the gaps between TiO_2_ particles but also improve the particle size distribution of cementitious materials and increase the compactness of colloids.

**Figure 5:**
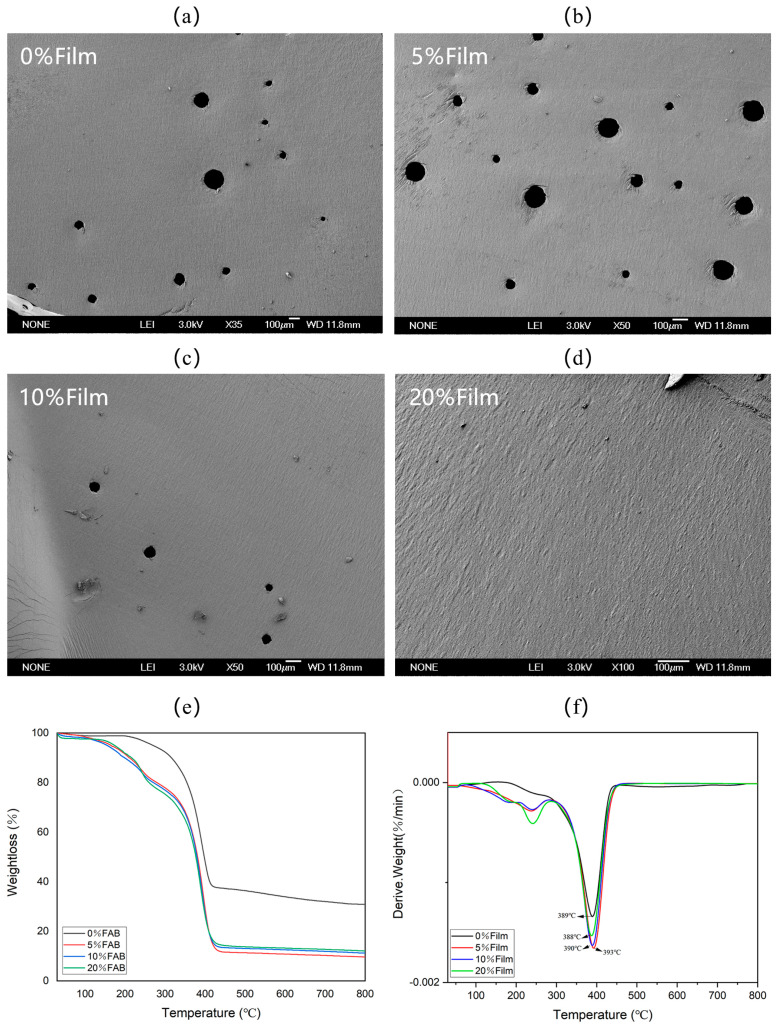
Microscopic morphology of (**a**) 0%Film; (**b**) 5%Film; (**c**) 10%Film and (**d**) 20%Film. (**e**) thermogravimetric analysis of the films; (**f**) comparison of thermal degradation rates of the films.

Figure 5a–d show the microscopic morphology of the FA surface coating, from which it can be seen that with the increase in the content of the color change agent, the pores on the surface of the coating are gradually reduced. When the content of the color change agent is 20%, there is no porosity on the surface of the coating; this is presumed to be due to the small size of the color change agent, which destroys the surface tension of the bubbles, making them break quickly, and plays the role of an antifoam agent. Figure 5e shows the TG graphs of the coatings with four different color change agent contents. Among them, the period between 203 and 250 °C is when the evaporation of water occurs in the sample, and the thermal degradation after that is attributed to the degradation of organic substances, such as acrylic resin, and the dissolution of NaOH on the sample surface. The thermal degradation after 410 °C is attributed to the crystalline transformation of TiO_2_ from anatase to rutile. At 800 °C, the remaining mass of 0%Film is the highest, reaching 30%, and the remaining material is mainly undecomposed inorganic matter and TiO_2_. The thermal degradation of the remaining samples with different percentages of the color change agent is similar to that of 0%Film, but it is not as thermally stable as that of 0%Film, and evaporation of the bound water started at 97 °C. The thermal degradation of 20%Film at 800 °C is attributed to the degradation of the TiO_2_ and the dissolution of the NaOH. At 800 °C, 20%Film has the highest residual mass of 12%, followed by 10%Film with 11% residual mass, and finally 5%Film with 9% residual mass, which is attributed to the higher thermal stability of the color change agent. Figure 5f shows that 0%Film has the slowest degradation rate, and with the addition of the color change agent, 5%Film has the fastest degradation rate, with the highest degradation temperature point at 393 °C. The sample with the slowest degradation rate is 20%Film, which suggests that the content of the color change agent has a positive effect on the thermal stability of the coatings, but it speeds up the decomposition rate of the coatings as comparing to the TiO_2_ coatings without the addition of the color change agent. However, the decomposition temperatures of all samples were over 100 °C, which meets the service temperature conditions for processing into jewelry.

**Figure 6:**
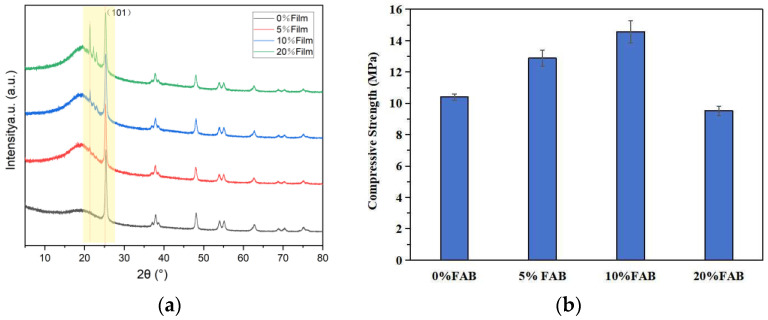
(**a**) XRD pattern of surface coating; (**b**) compressive strength test of FAB after coating treatment.

Figure 6a shows the X-ray diffraction results of the surface coatings. All samples show the same characteristic peaks, indicating that the color change agent is physically bonded to the other components. A strong diffraction peak occurs near 25°, which is a typical characteristic peak of TiO_2_ and corresponds to a crystallographic index of 101, which is related to the structure of the anatase phase of TiO_2_. The intensity of the 101 peak decreases slightly with the increase in the content of the color change agent, and a sharp peak appears near 21°. The peak around 21° is usually considered to be that of amorphous carbon, and the appearance of such a peak indicates that the carbon atoms in the material are arranged in a disordered or poorly ordered manner. The above results indicate that the discoloration agent may have an effect on the crystallinity of the surface coating, and the decrease in crystallinity may affect the mechanical strength of the material. Figure 6b shows the compressive strength test of FAB after coating treatment, and the results show that the compressive strength is increased when the amount of color change agent added does not exceed 10%. However, when the color change agent content exceeds 20%, the compressive strength of the samples decreases to 9.5 MPa. Among them, the compressive strength of 10%FAB is the highest, which reaches 14.6 MPa, exceeding that of F6S4, which is 10.1 MPa, suggesting that when the color change agent is controlled in a reasonable range, it has a positive effect on the overall compressive performance.

**Figure 7:**
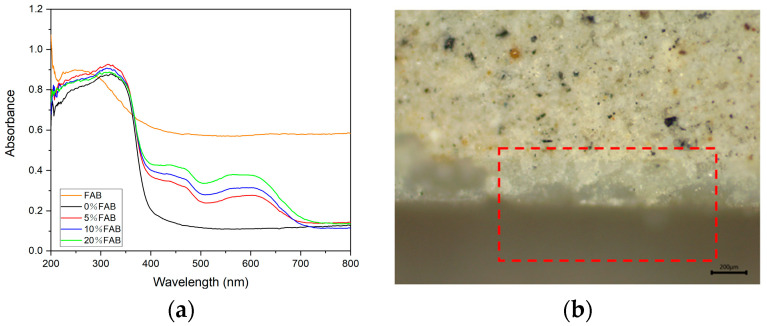
(**a**) UV-Vis absorption spectrum of FAB; (**b**) Microstructure of FA composite.

UV radiation is a part of solar radiation, and prolonged exposure to UV radiation may cause aging on the surface of necklace products, reducing the service life of the necklace. The wavelength range can be divided into UVA (320~400 nm), UVB (275~320 nm), and UVC (200~275 nm); because most of the UVC through the ozone layer has been absorbed, necklaces that are outdoors will only be subjected to ultraviolet radiation in the UVA and UVB wavelength regions, so the average absorbance of samples in the 275~400 nm range must be found to assess the necklace’s ultraviolet shielding ability. Figure 7a shows that that uncoated FAB has an all-optical absorption capacity, and the UV absorption intensity in the range of 275~400 nm is lower than that of the other coated treatments. Among the coated treatments, 5%FAB has the strongest UV absorption capacity, followed by 10%FAB. However, in the visible region, the absorbance is directly proportional to the content of the color change agent, which is attributed to the fact that when the content of the color change agent is low, the higher content of TiO_2_ in the surface coating provides better UV resistance. The bonding condition of the 10%FAB surface coating and the internal FA composite was observed by microscope (Figure 7b): the bonding between the surface coating and the internal composite was very dense, and there was no fracture at the bonding area, which is important for ensuring the dimensional stability of the surface coating.

**Figure 8:**
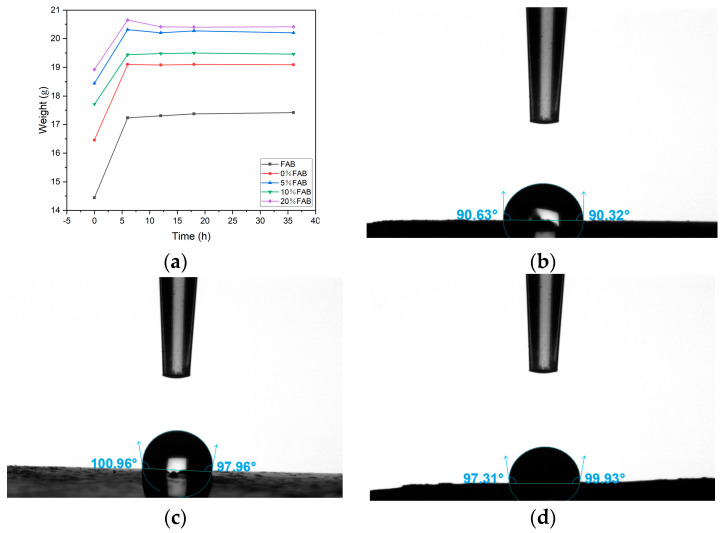
(**a**) Water absorption curve of FAB; (**b**) water contact angle of 5%FAB; (**c**) water contact angle of 10%FAB; (**d**) water contact angle of 20%FAB.

Figure 8 shows the water absorption curves of the samples, from which it can be seen that all the samples show an increase in weight in the first 6 h. Among them, FAB shows the strongest weight gain curve and still shows an increasing trend after the 35 h. This is attributed to the high internal porosity of FAB without UV coating, where moisture can penetrate into the specimen faster. Among the coating-treated FAB samples, 0%FAB shows the fastest rate of weight gain in the first 6 h. This may be related to the fact that TiO_2_ has a certain degree of water absorption, and the rate of water absorption decreases accordingly with the decrease in the percentage of TiO_2_. Using Table 2, the weight gain rate of five samples can be calculated as 20.6%, 15.9%, 9.5%, 9.8%, and 7.9%, respectively. The weight gain rate is the highest in FAB and 0%FAB, and the weight gain rate of the samples decreases rapidly after the addition of the color change agent: the weight gain rate of 20%FAB is the smallest, which indicates that the addition of the color change agent can improve the water repellency of FAB. Figure 8b–d show the water contact angles of 5%FAB, 10%FAB, and 20%FAB. The water contact angles of all samples were greater than 90°, among which 10%FAB has the largest water contact angle, and this hydrophobic surface plays an important role in improving the dimensional stability of FA composites.

**Figure 9:**
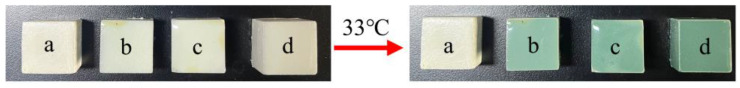
Color change effect of (**a**) 0%FAB; (**b**) 5%FAB; (**c**) 10%FAB; (**d**) 20%FAB.

In summary, F6S4 was selected as the substrate for the preparation of FA-based necklaces. In order to improve its dimensional stability and aesthetics, the surface of F6S4 was coated using acrylic resin doped with a color-changing agent, and when the ambient temperature exceeded 33 °C, the color of the FAB surface gradually deepened with the increase in the discoloration agent content (Figure 9). Among the samples, 10%FAB not only has a better surface color but also has better thermal stability, ultraviolet light absorption capacity, superhydrophobicity, and mechanical properties, so the 10%FAB was chosen as the basic material for the jewelry design.

### 3.2. Design Application of FAB

The 10%FAB sample was used as the design material, which was applied to the design of the necklace. The production of the necklace adopted the ancient Chinese traditional “gold inlaid jade” technology. In Chinese traditional culture, the combination of gold and jade has rich cultural connotations and auspicious symbols. They symbolize nobility and purity, honor, and good fortune and are also the symbol of love and marriage. With the improvement of modern aesthetic levels and to meet the consumption wishes of different consumer groups, influenced by the Western gemstone jewelry culture, the materials and processes used in gold and jade jewelry have changed, and jade jewelry has begun to be inlaid with 925 silver and other precious metals, so that the metal and the jade have different contrasting effects in material and color, creating a different visual experience. This study used FAB for jewelry design based on traditional Chinese craftsmanship, following the following design elements.

#### 3.2.1. Functional Design

FA-based color-changing materials have strong plasticity and light weight to meet the needs of wearable jewelry design, and the advantages of good wear resistance, water resistance, and drop resistance provide the basis for long-lasting wearable jewelry. Based on the characteristics of FA-based color-changing material, it is suitable to polish it into geometric elements applied in jewelry design, drawing on the traditional inlay process of combining gold and jade, but using waste FA material after modification. Through the use of new jewelry materials over traditional jewelry materials, traditional oriental craftsmanship, and the integration of new materials and innovation, bold material breakthroughs and innovations for jewelry design provides more possibilities, enriching the form of jewelry design and providing the basis for long-lasting wear. 

#### 3.2.2. Color Performance

**Figure 10:**
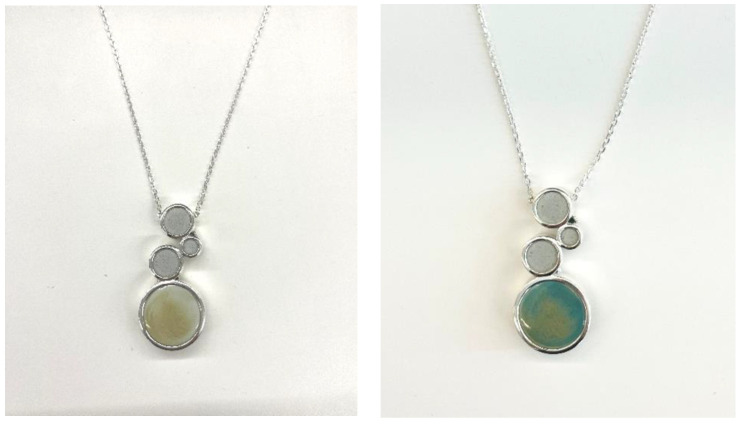
Color change effect of FA-based necklace.

The original color of the FA-based color-changing material is grey, and the grey tone of the color brings a sense of simplicity and sophistication. With the grey metal, the collision of different materials with the same hue can not only show the simple and unique high-level texture, but also highlight the sense of modern fashion. The change in color at different temperatures makes the work fresher and more interesting. Figure 10 shows the color-changing effect of the FA necklace. As can be seen in the figure, the color-changing material changes from gray to dark green after the temperature rises, presenting a jade-like effect, which increases the interest of the necklace.

#### 3.2.3. Modeling Design

The design of the necklace is based on purely decorative geometric elements, and the circle is chosen as the basic design element. One of the most basic geometric shapes, the circle does not have a clear starting point or end point and symbolizes rebirth, just like the generation of FA, which gives expression to the emotion of nirvana and the rebirth of the material.

The circle has a natural sense of flow and is a very flexible and diverse element. Different sizes of circle can be arranged and combined with a minimalist design to show the pure beauty of the jewelry and better highlight the natural texture of the material, without excessive decoration but exuding a unique charm. The external inlay of the delicate metal makes the whole piece more powerful and futuristic.

**Figure 11:**
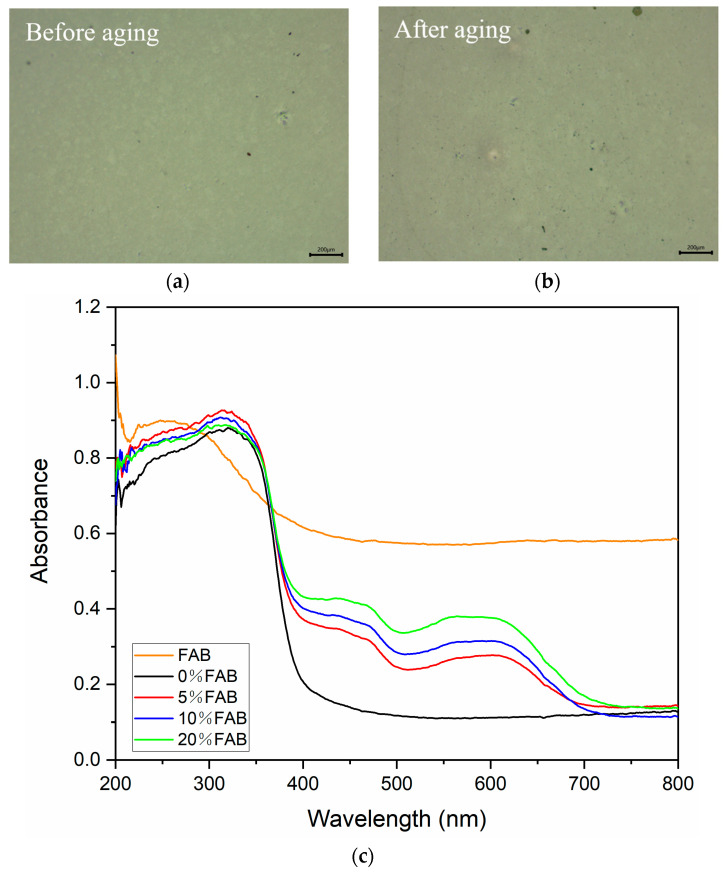
(**a**) Surface of necklaces before UV aging; (**b**) surface of necklaces after UV aging; (**c**) comparison of UV light absorption capacity of necklaces before and after aging.

UV aging experiments were used on the necklace in the actual environment to observe the long-term photothermal response, simulating the sample in actual use. The test was conducted at 40 °C, using 365 nm UV lamp irradiation (ZLUV LAM, Shenzhen, China), with the sample placed 400 mm from the light source, with an aging time of 100 h. It can be seen that there are no cracks, bubbles, folds, etc., on the surface of the samples before or after the aging experiment (Figure 11a,b). The UV spectroscopy detection reveals that the all-optical light absorption capacity of the necklace surface is elevated after prolonged UV aging (Figure 11c), which is attributed to the deepening of the color of the sample surface after prolonged exposure to light. In addition, after a long period of UV irradiation, the electrons in the valence band of TiO_2_ within the FAB surface coating are excited to the conduction band to form hole–electron pairs, and these hole–electron pairs are highly reactive, which strengthens the light absorption degree of TiO_2_ in the UV region. Therefore, the FA-based necklaces prepared in this study have good aging resistance and can be used in outdoor environments for a long time.

## 4. Conclusions

The low utilization rate of fly ash, as a waste product of coal-fired power generation, exerts enormous pressure on the environment. In order to improve the high-net-value utilization of fly ash, this study took fly ash as a research object and prepared fly-ash-based composite material FAB by blending it with coal slag, TiO_2_, sodium silicate, and other materials. Then, FAB was applied to jewelry design through the traditional Chinese “gold inlaid jade” process. The main research conclusions of this study are as follows.

After blending fly ash and slag, an alkaline activator with a total mass of 10% was added. When the proportion of fly ash was 60%, the sodium silicate in the alkaline activator reacted with free CaO, MgO, and other substances in the fly ash to form hydrated silicate colloids. The compressive strength of fly-ash-based composite materials can reach 10.1 MPa. In order to protect the surface of fly-ash-based composite materials, an anti-ultraviolet coating with thermochromic function was obtained by blending acrylic resin, TiO_2_, and thermosensitive color-changing agent. This was applied to the surface of the fly-ash-based composite materials. When the mass fraction of the color-changing agent was 10%, the prepared 10%FAB not only had good surface color but also had good thermal stability, UV absorption ability, superhydrophobicity, and mechanical properties. Using the traditional Chinese technique of “gold inlaid with jade”, 10%FAB was made into a necklace. The necklace not only has modern design aesthetics but also turns green when the temperature exceeds 33 °C, meeting the unity of functionality and aesthetics. After a long period of UV aging experiments, the necklace did not show any wrinkles, bubbles, or other phenomena. This study provides new ideas for the “optimal use of inferior materials” in solid waste, which is of great significance for promoting global sustainable development.

## Figures and Tables

**Table 1:** Chemical reagents and their properties.

Sample Name	Performance	Chemical Abbreviations	Manufacturer
Fly ash	15–30 μm, specific surface area 430 m^2^/kg, density 2.42 g/cm^3^	FA	Hebei Huadian Shijiazhuang Thermal Power Co., Ltd., Shijiazhuang, China
Slag	30–60 μm	/	Hebei Huadian Shijiazhuang Thermal Power Co., Ltd., Shijiazhuang, China
Sodium silicate	/	Na_2_O-nSiO_2_	Shanghai Aladdin Biochemical Technology Co., Ltd., Shanghai, China
Sodium hydroxide	/	NaOH	Tianjin Baima Technology Co., Ltd., Tianjin, China
Titanium dioxide	20–30 nm	TiO_2_	Shanghai Aladdin Biochemical Technology Co., Ltd., Shanghai, China
Acrylic resin	/	AR	Shanghai Aladdin Biochemical Technology Co., Ltd., Shanghai, China
Color change agent	Color change temperature 33 °C	/	Shenzhen Phantom Color Change Technology Co., Shenzhen, China

**Table 2:** Water absorption weight of FAB over time.

Sample	0 h	6 h	12 h	18 h	36 h
FAB	14.44 g	17.23 g	17.30 g	17.37 g	17.41 g
0%FAB	16.46 g	19.10 g	19.08 g	19.10 g	19.09 g
5%FAB	18.44 g	20.31 g	20.20 g	20.27 g	20.20 g
10%FAB	17.71 g	19.44 g	19.48 g	19.50 g	19.46 g
20%FAB	18.92 g	20.65 g	20.41 g	20.40 g	20.41 g

## Data Availability

The original contributions presented in this study are included in the article. Further inquiries can be directed to the corresponding author.
